# Clinical Report from Medical Wards of Mercy Hospital

**Published:** 1868-12

**Authors:** N. S. Davis

**Affiliations:** Professor of Clinical Medicine, etc.


### CLINICAL REPORT FROM MEDICAL WARDS OF MERCY HOSPITAL.

Service of N. S. DAVIS, M.D., Professor of Clinical Medicine, etc. Reported by W. A. BARSTOW.

GENTLEMEN:—This young man, occupying the bed before you, has an affection of the glands of the groin. At first, it would seem as though it were due to inoculation with a specific poison. This is not the case, however, as the patient has not had any form of syphilitic disease, at least for several years. The enlargement of the glands has come on gradually, affording him little pain, except when attempting to walk. There are evidently several glands enlarged on left side, and some on opposite side.

These glands are apt to enlarge when the patient has been exposed to cold or wet. When due to this cause, the inflammation usually develops suddenly, and subsides in two or three days, without treatment. In other instances, they arise from a sore on the foot, an ingrowing toe-nail, or perhaps some sore midway between the ankle and knee; the tenderness, in such cases, extending along the course of the lymphatics to the groin. The most frequent cause, however, of this form is from a sore on the foot. Without these conditions, we will find patients who are pale, cachectic, blood-impoverished, tissues not well nourished, with indications of diseased lymphatic glands in more than one situation: sometimes in the neck, extending down to the clavicle; sometimes extending down along the larger bronchial tubes, creating great distress, cough, and wheezing.

I once had a lady under my care whose bronchial glands were so enlarged that she could endure but little exercise. If she went up-stairs quickly, it would cause her to wheeze terribly.

Sometimes we meet with chronic enlargements in the groin, along the edge of the pelvis; and I have no doubt but what these sometimes extend into the abdomen.

When associated with a scrofulous or tuberculous diathesis, the prognosis is uncertain, and the treatment often protracted. I think this patient is inclined to the above diathesis, as the pallor, thin flesh, and rather narrow chest, would indicate. He is easily made sick at the stomach. Bowels inclined to be constipated. If you examine the tumor in the left groin, you find it oblong, parallel with Poupart's ligament, and made up of a chain of enlarged and indurated glands. They are but little sensitive, and uniformly hard, except a small soft place on the most prominent one to the left, which feels like a small spot of suppuration.

Locally, we will keep the enlarged glands covered with a linseed poultice, wet up with an infusion of aconite leaves. To improve the general health, and render the digestive and assimilative functions more active, and thereby aid in causing resolution in the enlarged glands, we will give him ten drops of the following solution, before each meal, in a wineglassful of water:—

℞. Iodine, ---------------------------- 10 grs.

Iodide Potassæ, ------------------ ʒj.

Water, ---------------------------- ℥j.

Also one of the following pills at bedtime each night:—

℞. Ext. Hyoscyamus, --------------- 30 grs.
Sulph. Ferri, --------------------- 30 grs.
Pulv. Aloes, ---------------------- 30 grs.
Blue Mass, ----------------------- 15 grs.
Ext. Nux Vom., ------------------ 15 grs.

Mix. Divide 30 pills.

It may be that the soft point in the left groin will require opening. But will defer it until it is farther advanced, and it may be found to recede without any external discharge.

The attention of the Class was directed briefly to the progress of two other cases of disease in the same ward.

Note.—The foregoing treatment was continued ten days, during which the enlarged glands nearly disappeared, and the general health of the patient was much improved.

